# Transcriptome analyses suggest minimal effects of Shank3 dosage on directional gene expression changes in the mouse striatum

**DOI:** 10.1080/19768354.2019.1595142

**Published:** 2019-04-12

**Authors:** Yeunkum Lee, Hyojin Kang, Chunmei Jin, Yinhua Zhang, Yoonhee Kim, Kihoon Han

**Affiliations:** a Department of Neuroscience, College of Medicine, Korea University, Seoul, South Korea; b Department of Biomedical Sciences, College of Medicine, Korea University, Seoul, South Korea; c Division of National Supercomputing, KISTI, Daejeon, South Korea

**Keywords:** Shank3 dosage, striatum, transcriptome

## Abstract

Both deletions and duplications of the SH3 and multiple ankyrin repeat domains 3 (*SHANK3*) gene, encoding excitatory postsynaptic scaffolds, are causally associated with various brain disorders, suggesting that proper Shank3 dosage is critical for normal brain development and function. In addition to its well-established synaptic functions, recent studies have suggested that Shank3 can also affect gene expression in the nucleus. However, it has not been investigated whether there are a group of genes whose directional expression is regulated in a Shank3 dosage-dependent manner (i.e. showing opposite changes in expression following Shank3 reduction and overexpression). This is an important issue to be examined for better understanding why neuronal development and function are sensitive to Shank3 dosage, and how much transcriptional changes contribute to neuronal phenotypes affected by Shank3 dosage. To examine this, we performed transcriptome analyses on the striatum of *Shank3* heterozygous and knock-out mice, which identified three and 17 differentially expressed genes, respectively. We then compared the results to those of our previous striatal transcriptome analysis of *Shank3* overexpressing mice and identified 31 candidate genes showing directional expression changes in a Shank3 dosage-dependent manner. However, overall, their Shank3 dosage-dependent fold changes were very subtle (average of absolute log2(fold change) was 0.139). Meanwhile, the gene set enrichment analyses of the striatal transcriptome suggested that Shank3 dosage may affect anchoring junction-related functions. Taken together, these results suggest that Shank3 dosage minimally affects directional gene expression changes in the mouse striatum.

## Main text

The SH3 and multiple ankyrin repeat domains 3 (*SHANK3*) gene encodes core scaffolding proteins organizing the macromolecular protein complex of the neuronal excitatory postsynapses (Sheng & Kim 2000). Both deletions and duplications of the chromosomal region containing the *SHANK3* gene are causally associated with various neurodevelopmental and neuropsychiatric disorders, including autism spectrum disorders, schizophrenia, and bipolar disorder (Han et al. 2013; Monteiro & Feng 2017). Therefore, proper Shank3 dosage is critical for normal brain development and function (Toro et al. 2010). Mechanistically, biochemical, morphological, and functional changes of neuronal excitatory synapses in several brain regions were identified from both *Shank3* knock-out (KO) and overexpressing transgenic (TG) mice (Monteiro & Feng 2017).

Notably, beyond the synaptic changes, recent studies have suggested that Shank3 can also directly and indirectly affect gene expression in the nucleus, which may contribute to the neuronal pathophysiology of *SHANK3*-associated brain disorders. For example, Shank3 undergoes synapse-to-nucleus shuttling in an activity-dependent manner, which affects transcription of several genes (Grabrucker et al. 2014). In addition, synaptic Shank3-deficiency leads to increased nuclear localization of *β*-catenin, a Shank3-binding protein, which causes histone deacetylase 2 (HDAC2)-dependent transcriptional changes (Qin et al. 2018). Furthermore, our group performed transcriptome analyses (RNA-sequencing (RNA-seq)) in several brain regions of *Shank3* TG mice, including the striatum (Lee, Kim et al. 2017), medial prefrontal cortex (Jin, Kang, Ryu et al. 2018), and hypothalamus (Jin, Kang, Kim et al. 2018), and identified several differentially expressed genes (DEGs) from each brain region compared to wild-type (WT) mice. Even with this evidence, however, whether there are a group of genes whose expression is regulated in a Shank3 dosage-dependent manner (i.e. genes showing opposite changes in expression following Shank3 reduction and overexpression) has not been directly investigated. We reasoned that this is an important issue to be examined for better understanding why neuronal development and function are sensitive to Shank3 dosage, and how much transcriptional changes contribute to neuronal phenotypes affected by Shank3 dosage.

**Figure 1.**
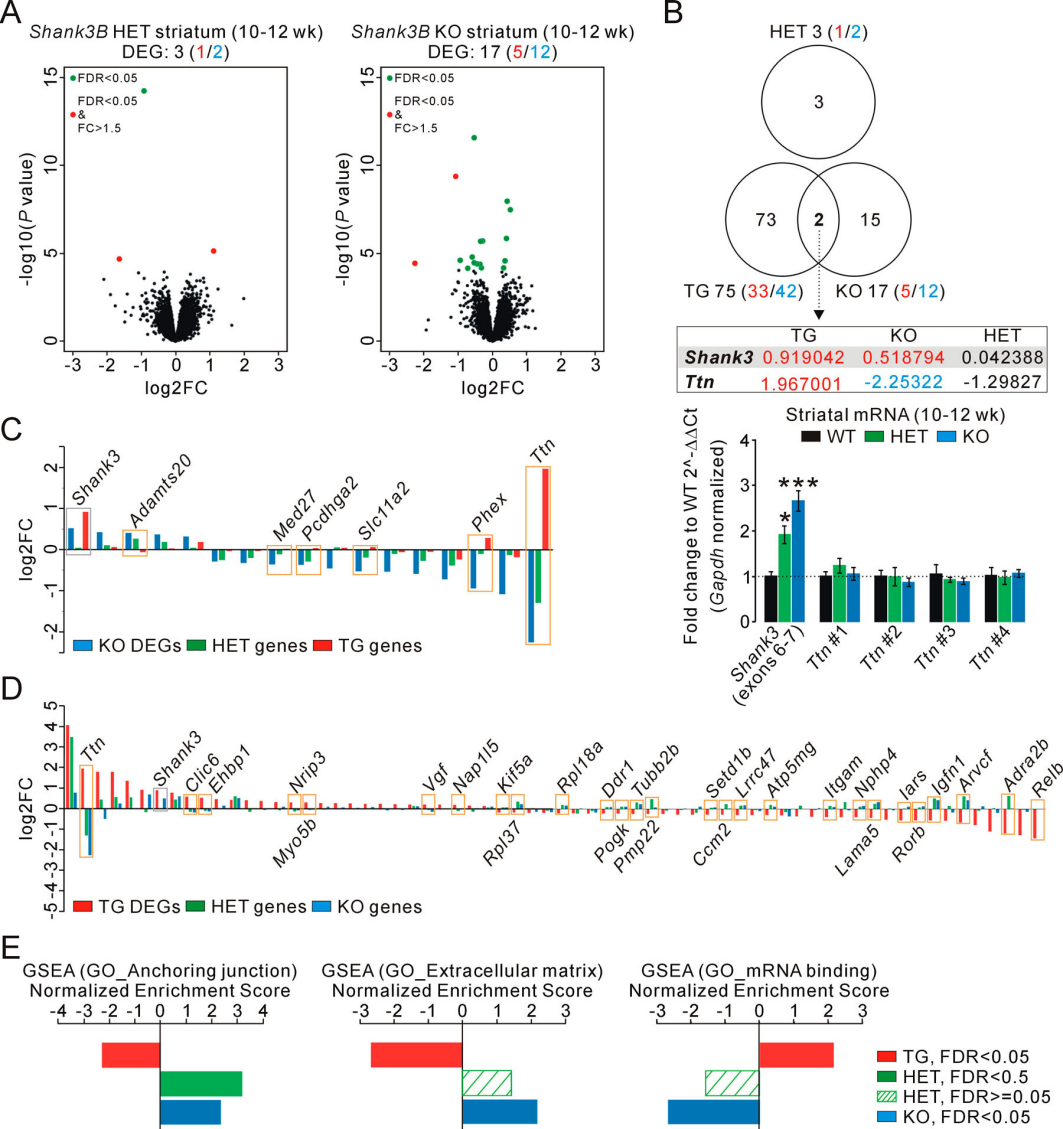
Transcriptome analyses of the *Shank3B* heterozygous and knock-out striatum and comparison with that of the *Shank3* overexpressing striatum. (A) Volcano plots for the striatal RNA-sequencing (RNA-seq) analyses of adult *Shank3B* heterozygous (HET) and knock-out (KO) mice. Differentially expressed genes (DEGs), defined by false discovery rate (FDR) < 0.05, are shown as green (FC < = 1.5) and red (FC > 1.5) circles. FC, fold change; wk, weeks. (B) The Venn diagram shows the number of common DEGs among the KO, HET, and TG striatal RNA-seq analyses. For the common DEGs (*Shank3* and *Ttn*), the log2FC values for each genotype are shown. The bar graph shows the qRT-PCR results for *Shank3* and *Ttn* (four different primer sets) in the HET and KO striatum (*n* = 4 animals per each genotype). (C) The bar graph shows log2FC values for the 17 DEGs of the KO striatum and for the corresponding genes in the HET and TG striatum. The *Shank3* and six candidate genes are highlighted. (D) The bar graph shows log2FC values for the 75 DEGs of the TG striatum and for the corresponding genes in the HET and KO striatum. The *Shank3* and 27 candidate genes are highlighted. (E) The bar graphs show opposite directional normalized enrichment score (NES) values of the gene set enrichment analysis (GSEA) for the TG, HET, and KO striatal transcriptome on the three gene ontology (GO) gene sets. Data are presented as mean ± SEM. *P < 0.05; ***P < 0.001 (One-way ANOVA, Tukey’s post-hoc test).

We performed RNA-seq analysis in the striatum of adult *Shank3B* (exons 13–16 of *Shank3* gene are targeted) (Peca et al. 2011) heterozygous (HET), KO, and WT littermate mice and compared the results with that of our previous striatal RNA-seq analysis of age-matched *Shank3* TG mice (Lee, Kim et al. 2017; Jin, Kang, Ryu et al. 2018). We focused on the striatum because *Shank3* is highly enriched in the brain region, and because *Shank3B* KO mice display neuronal defects mainly in the striatum (Peca et al. 2011). Compared to the striatum of *Shank3* TG mice (75 DEGs; 33 up-regulated and 42 down-regulated), there was much less number of DEGs from both *Shank3B* HET (three DEGs; one up-regulated and two down-regulated) and KO striatum (17 DEGs; five up-regulated and 12 down-regulated) (Figure 1(A); Supporting material 1(Table 1 and 2)). When the three DEG lists were compared, only two genes (*Shank3* and *Titin* [*Ttn*]) were common between the TG and KO striatum, and there was no overlap between the HET and either TG or KO striatum (Figure 1(B)). Unexpectedly, similar to the TG striatum, total *Shank3* transcripts were increased in the KO striatum (Figure 1(B)). This was due to increased expression of the non-targeted *Shank3* exons (mostly exons 1–12, possibly as a compensatory response) (Supporting material 1(Figure 1)), which was confirmed by qRT-PCR experiments with *Shank3* primers against exons 6–7 (Figure 1(B)).

The other common DEG, *Ttn*, was up- and down-regulated in the TG and KO striatum, respectively (Figure 1(B)). Moreover, *Ttn* expression showed a trend of decrease in the HET RNA-seq analysis. Therefore, *Ttn* could be a candidate gene whose striatal expression is regulated in a Shank3 dosage-dependent manner. However, qRT-PCR validations of the HET and KO striatum with four independent primer sets targeting different exons of *Ttn* did not show its expression change relative to the WT striatum (Figure 1(B)). Therefore, we concluded that *Ttn* was a false positive DEG of the RNA-seq analyses, which may be caused by the large number of *Ttn* exons (> 300 encoding a protein of 4,200 kDa) and its low expression level in the striatum (i.e. low basemean value [ < 40] from the transcriptome analysis, Supporting material 1(Table 2)).

We further examined whether we could identify additional candidate genes, other than *Ttn*, with less stringent criteria. Specifically, among the 17 DEGs of the KO striatum, we selected those genes satisfying both showing the same directional expression changes in the KO and HET, and showing opposite directional expression changes in the KO and TG (regardless of significance). For example, protocadherin gamma subfamily A 2 (*Pcdhga2*) gene (down-regulated DEG in KO) was selected because it was down- and up-regulated in the HET and TG striatum, respectively. We identified five genes other than *Ttn* among the 17 DEGs of the KO striatum (Figure 1(C)). However, the fold change (FC) values of them in the TG striatum were very low (average of absolute log2FC, 0.093) (Supporting material 1(Table 3)). In a similar manner, 26 genes other than *Ttn* were identified among the 75 DEGs of the TG striatum, but their FC values in the KO striatum were also very low (average of absolute log2FC, 0.148) (Figure 1(D); Supporting material 1 (Table 4)). Gene ontology (GO) analysis on the 31 genes did not reveal any significant term (data not shown).

Next, we performed gene set enrichment analysis (GSEA) of the HET and KO striatal transcriptome to understand molecular signatures based on broader expression changes rather than specific DEGs. We then compared the results with our previous GSEA of the TG striatal transcriptome (Lee, Kim et al. 2017; Jin, Kang, Ryu et al. 2018) to identify significant GSEA terms with opposite directional normalized enrichment scores (NESs) in the KO and TG striatum. We identified three terms from the GO category, ‘anchoring junction’, ‘extracellular matrix’, and ‘mRNA binding’ (Figure 1(E)). Notably, for all three terms, the HET NES values showed the same directions as those of KO, and the ‘anchoring junction’ term was also significantly enriched for the HET striatal transcriptome (Figure 1(E)). Since GSEA often reflects downstream transcriptional responses, rather than direct regulation, to changes in the cellular state, these results suggest that Shank3 dosage may affect anchoring junction-related functions in the mouse striatum, which requires further investigations to be validated.

In conclusion, our results suggest minimal effects of Shank3 dosage on directional gene expression changes in the mouse striatum. In other words, at least in the striatum, Shank3 reduction and overexpression affect different group of genes with different degrees. Therefore, transcriptional changes, compared to synaptic changes, may have much less contribution to the striatal neuronal phenotypes sensitive to Shank3 dosage. Nevertheless, we cannot exclude the possibility that gene expression in other brain regions, such as the medial prefrontal cortex, may be more sensitive to Shank3 dosage than that in the striatum (Jin, Kang, Ryu et al. 2018; Qin et al. 2018). Moreover, considering the developmental functions of Shank3, Shank3 dosage may have stronger effects on directional gene expression changes at early developmental stages. Intriguingly, we found and confirmed increased expressions of the non-targeted *Shank3* exons in the *Shank3B* Het and KO striatum. We speculate that synaptic loss of Shank3 induces transcription of specific *Shank3* isoforms via certain mediators, such as *β*-catenin (Qin et al. 2018), which translocate from synapse to the nucleus.

## Materials and methods

### Mice

The *Shank3B* HET and KO mice used in this study have been described previously (Peca et al. 2011). The male WT, HET, and KO mice were bred and maintained in a C57BL/6J background according to the Korea University College of Medicine Research Requirements, and all the experimental procedures were approved by the Committees on Animal Research at the Korea University College of Medicine (KOREA-2016-0096). The mice were fed and had access to water *ad libitum* and were housed under a 12-h light–dark cycle.

### RNA sequencing and analysis

The mice (10–12-week-old male WT, HET, and KO) were deeply anesthetized with isoflurane and decapitated. The striatum was dissected from each brain, immediately placed in RNAlater solution (Ambion), and stored at 4°C overnight. The striatum from one mouse was used to make one RNA sample, and a total three sets of RNA samples (total three mice per each genotype) were processed for RNA sequencing. RNA extraction, library preparation, cluster generation, and sequencing were performed by Macrogen Inc. (Seoul, Korea). RNA samples for sequencing were prepared using a TruSeq Stranded mRNA LT Sample Prep Kit (Illumina) according to the manufacturer’s instructions. An Illumina’s HiSeq 2000 was used for sequencing to generate 101-bp paired-end reads. Raw data were submitted to the GEO (Gene Expression Omnibus) repository under accession number GSE124946.

Transcript abundance was estimated with Salmon (v0.9.1) (Patro et al. 2017) in Quasi-mapping-based mode onto the Mus musculus genome (GRCm38) with GC bias correction (–gcBias). Quantified gene-level abundance data was imported to R (v.3.6.0) with the tximport (Soneson et al. 2015) package and differential gene expression analysis was carried out using R/Bioconductor DEseq2 (v1.19.11) (Love et al. 2014). Normalized read counts were computed by dividing the raw read counts by size factors and fitted to a negative binomial distribution. The *P* values were first corrected by applying an empirical estimation of the null distribution using the R fdrtool (v.1.2.15) package and then adjusted for multiple testing with the Benjamini–Hochberg correction. Genes with an adjusted *P* value of less than 0.05 were considered as differentially expressed. Volcano plots were generated using the R ggplot2 (v.2.2.1) package.

The GO and Kyoto Encyclopedia of Genes and Genomes (KEGG) pathway analyses were performed using DAVID software (version 6.8) (Huang da et al. 2009). Mouse gene names were converted to human homologs using the Mouse Genome Informatics (MGI) database (http://www.informatics.jax.org/homology.shtml).

GSEA (http://software.broadinstitute.org/gsea) (Subramanian et al. 2005) was used to determine whether a *priori*-defined gene sets would show statistically significant differences in expression between *Shank3B* and WT mice. Enrichment analysis was performed using GSEAPreranked (gsea-3.0.jar) module on gene set collections H (Hallmark gene sets; 50 gene sets) and CP (KEGG; 186 gene sets) downloaded from Molecular Signature Database (MSigDB) v6.1 (http://software.broadinstitute.org/gsea/msigdb). GSEAPreranked was applied using the list of all genes expressed, ranked by the fold change and multiplied by the inverse of the *P* value with recommended default settings (1,000 permutations and a classic scoring scheme). The False Discovery Rate (FDR) was estimated to control the false positive finding of a given NES by comparing the tails of the observed and null distributions derived from 1000 gene set permutations. The gene sets with an FDR of less than 0.05 were considered as significantly enriched.

### RNA purification and qRT-PCR

Real-time quantitative reverse transcription PCR (qRT-PCR) was performed as described previously (Kim et al. 2016; Lee, Zhang et al. 2017). Briefly, total RNA was extracted from the straitum of WT and *Shank3B* mice using an miRNeasy Mini Kit (Qiagen) according to the manufacturer’s instructions. Two micrograms of total RNA was used for cDNA synthesis using iScript™ cDNA Synthesis Kit (Bio-Rad). Target mRNAs were detected and quantified by a real-time PCR instrument (CFX96 Touch, Bio-Rad) using SYBR Green master mix (Bio-Rad). The results were analyzed using the comparative Ct method normalized against the housekeeping gene *Gapdh*. The primer sequences for real-time PCR are as follows:

*Gapdh* forward 5′ GGCATTGCTCTCAATGACAA 3′,

   reverse 5′ CCCTGTTGCTGTAGCCGTAT 3′

*Shank3* forward 5′ TGGTTGGCAAGAGATCCAT 3′,

   reverse 5′ TTGGCCCCATAGAACAAAAG 3′

*Ttn* #1(exon 28) forward 5′ GACACCACAAGGTGCAAAGTC 3′

   reverse 5′ CCCACTGTTCTTGACCGTATCT 3′

*Ttn* #2 (exon 275) forward 5′ CTACGTGGTAGAAAAGCGAGAAA 3′

   reverse 5′ ACACCGTACTTGTTGACAGCC 3′

*Ttn* #3 (exon 232) forward 5′ CTCCAGCCAAAGACGGTGG 3′

    reverse 5′ GCAGTGAGAAGTTTATCGGGTTC 3′

*Ttn* #4 (exon 307) forward 5′ ACCAAAGAAGATAAGACCAGAG 3′

    reverse 5′ GACAATTCCAAACTCACCAC 3′
